# CySBGN: A Cytoscape plug-in to integrate SBGN maps

**DOI:** 10.1186/1471-2105-14-17

**Published:** 2013-01-16

**Authors:** Emanuel Gonçalves, Martijn van Iersel, Julio Saez-Rodriguez

**Affiliations:** 1, EMBL - European Bioinformatics Institute, Cambridge, UK

## Abstract

**Background:**

A standard graphical notation is essential to facilitate exchange of network representations of biological processes. Towards this end, the Systems Biology Graphical Notation (SBGN) has been proposed, and it is already supported by a number of tools. However, support for SBGN in Cytoscape, one of the most widely used platforms in biology to visualise and analyse networks, is limited, and in particular it is not possible to import SBGN diagrams.

**Results:**

We have developed CySBGN, a Cytoscape plug-in that extends the use of Cytoscape visualisation and analysis features to SBGN maps. CySBGN adds support for Cytoscape users to visualize any of the three complementary SBGN languages: Process Description, Entity Relationship, and Activity Flow. The interoperability with other tools (CySBML plug-in and Systems Biology Format Converter) was also established allowing an automated generation of SBGN diagrams based on previously imported SBML models. The plug-in was tested using a suite of 53 different test cases that covers almost all possible entities, shapes, and connections. A rendering comparison with other tools that support SBGN was performed. To illustrate the interoperability with other Cytoscape functionalities, we present two analysis examples, shortest path calculation, and motif identification in a metabolic network.

**Conclusions:**

CySBGN imports, modifies and analyzes SBGN diagrams in Cytoscape, and thus allows the application of the large palette of tools and plug-ins in this platform to networks and pathways in SBGN format.

## Background

The illustration of information in biology is frequently made using diagrams. Therefore, having a standard notation is very beneficial as it facilitates interpretation and forgoes the need for long and detailed explanations [1]. Motivated by this, a large community of biochemists, modellers and computer scientists proposed the Systems Biology Graphical Notation (SBGN) [1]. SBGN is an established effort for a standard graphical notation in biology. It leverages previous efforts, including Molecular Interactions Maps [2,3] and Kitano process diagrams [4,5], and extracts the best of graphical standard notations in other fields, such as Unified Modelling Language in software engineering.

SBGN is subdivided into three different and complementary languages: Process Description (PD), Entity Relationship (ER) and Activity Flow (AF). This subdivision allows SBGN to be accurate and assures an unambiguous representation [1]. PD focuses on the representation of temporal changes occurring between biochemical entities. All molecular processes and interactions and their results are depicted in PD diagrams. In contrast, the ER language depicts the effects among entities, emphasizing the influences that each entity has on each other and disregarding the transformations on itself (which are dealt with in PD diagrams). Finally, AF graphically shows the influence of activities such as inhibition or activation. To better represent these influences the diagram is simplified by ignoring biochemical details of the process and entity states, reducing the number of nodes when compared to an equivalent PD. These three languages together enable the users to represent all types of biological information from biochemical interaction maps to cellular signalling networks.

A graphical notation can only be shared and analysed if it is supported by network visualization tools. SBGN is currently supported by a list of 24 software packages (http://www.sbgn.org/), including CellDesigner [6], PathVisio [7] and VANTED [8,9]. Cytoscape [10,11], arguably the most popular tool in bioinformatics for visualization and analysis of biological networks, has support for the Systems Biology Markup Language (SBML) [12] natively and via the CySBML plug-in [13], and BioPAX [14] via the BiNoM plug-in [15]. However, it currently has no support for SBGN. The ability to import SBGN diagrams into Cytoscape would be very beneficial, as it would enable the use of all the network analysis features of Cytoscape and its plug-ins in SBGN maps. These include the analysis of the network properties (e.g. shortest paths, motif discovery), visualization of expression data, and pathway modelling.

Motivated by this, we have developed CySBGN, a new plug-in for Cytoscape that enables the full support of SBGN diagrams. CySBGN allows one to import and visualize SBGN diagrams stored in SBGN-ML format using the libSBGN library [16]. It is also possible to export small changes made in the SBGN diagram, such as node positions. Full integration with CySBML [13] and Systems Biology Format Converter [17] is available, allowing the user to automatically generate SBGN diagrams from SBML models. To validate the rendering of the diagrams with the plug-in, we tested it with a suite of SBGN diagrams that covers the three sub languages as well as all its shapes and connections. The plug-in applicability is also demonstrated by applying network analysis methods, in particular shortest path and motif discovery, from two different plug-ins in a PD diagram of the central plant metabolism.

## Implementation

CySBGN enables the importation of SBGN diagrams into Cytoscape and it is compatible with the latest version of Cytoscape, version 2.8.3. All the entity and relationship nodes shapes defined in SBGN specifications for each language are supported.

### 

#### Using libSBGN library and SBGN-ML format

**Figure 1:**
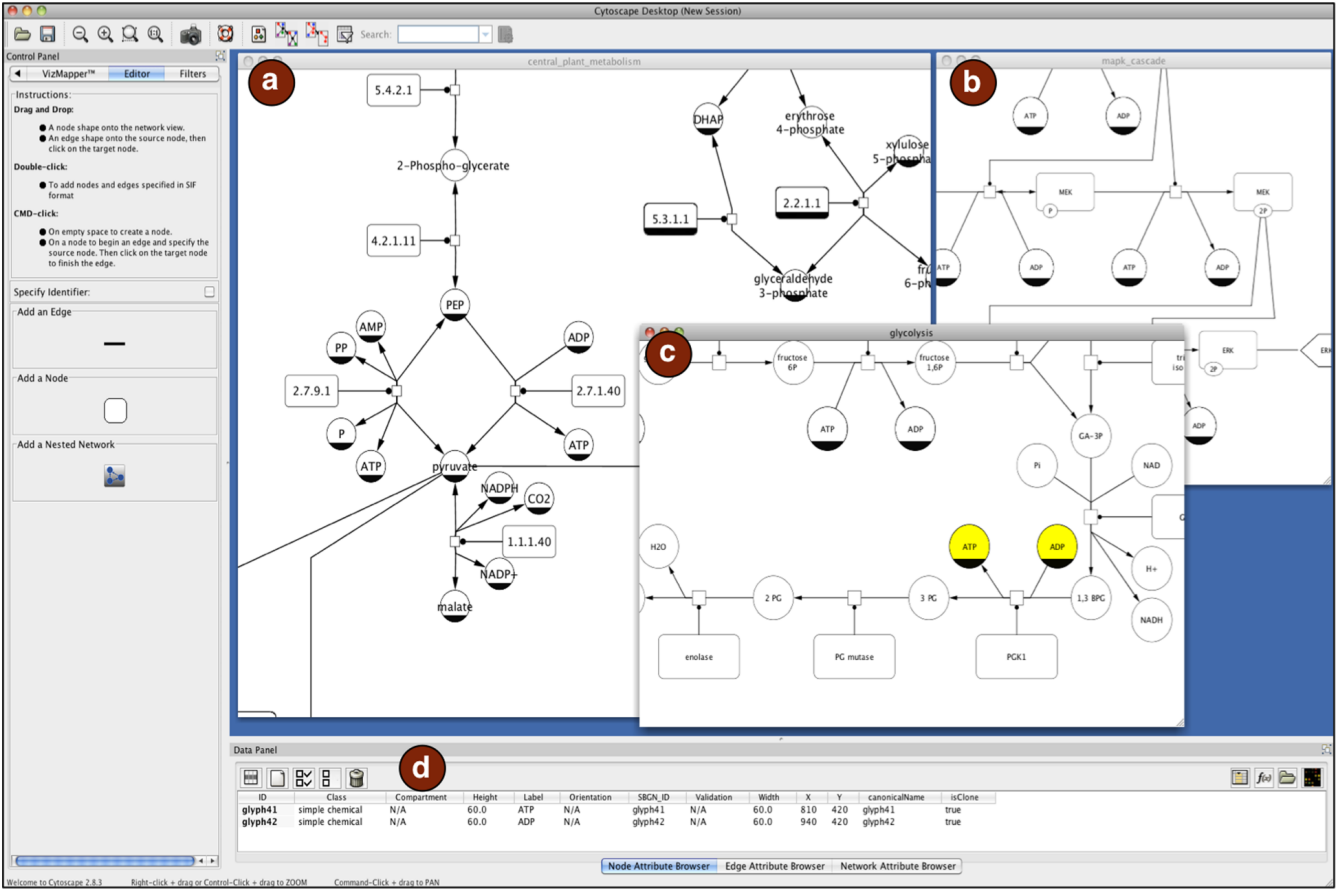
**CySBGN overview.** CySBGN overview. Diagram *(a)* shows a small part of the imported diagram of the central plant metabolism with 1324 nodes and 1322 edges. Diagram *(b)* depicts par of the imported MAPK cascade model. Diagram *(c)* represents the glycolysis metabolic pathway. Panel *(d)* is the Data Panel of Cytoscape where the user can check the node and edge attributes loaded from the SBGN-ML file. Both diagram *(a)* and *(b)* are part of the core validation tests of CySBGN. All these examples are part of the test examples of libSBGN and the examples of SBGN. All the test files are available on CySBGN project web page http://sourceforge.net/projects/cysbgn/.

The success of a widely used notation is also dependent on the digital storing format, since it determines how easily the notation can be shared and interpreted. Hence, a digital format should be easy to understand, capable of capturing accurately all the information and save it for further access. To face this challenge the libSBGN library and the SBGN-ML format were recently presented [16]. libSBGN is a Java library that provides an API to save, load, query and validate SBGN-ML files, that are structured as an extension of the XML file format. CySBGN makes use of these technologies to display PD, AF and ER diagrams in Cytoscape. The diagrams are loaded and then converted into Cytoscape’s network structure. In order to represent the SBGN graphical notation a VizMapper visual style is applied according to the attributes of the nodes and the edges (see Figure 1).

All the information from the SBGN-ML file used to create and display the SBGN diagram is stored as node and edge attributes. Therefore, information such as node type (e.g. phenotype, macromolecule), node width and edge type (e.g. stimulation, necessary-stimulation) is available to the user through the Data Panel in Cytoscape.

CySBGN stores in the SBGN-ML files the coordinates of all entities in the diagram, allowing the user to export a defined layout. Thus, one can use CySBGN to take advantage of the several Cytoscape automatic layout algorithms and then share the generated layout among other SBGN compliant tools.

#### Implementing SBGN in Cytoscape

**Figure 2:**
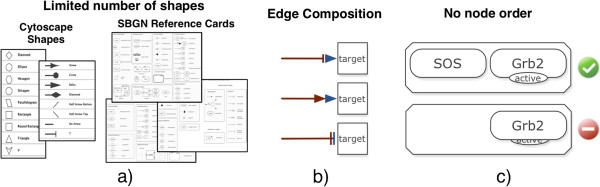
**Implementing SBGN in Cytoscape.** Rendering limitations. In picture (**a**) the reduced amount of node and edge shapes available in Cytoscape to represent all the entities present in the reference cards of SBGN is noticeable. Picture (**b**) illustrates how the SBGN edge shapes that are not supported in Cytoscape are obtained with CySBGN by an aggregation of supported edges shapes using an auxiliary and invisible node between them. The colour distinguishes the different types of nodes used to obtain the final edge shape. Picture (**c**) depicts how the random ordering of the nodes may affect the visualization of the diagram.

Some of the specifications of SBGN are directly representable in Cytoscape, while some need to be represented in different ways (see Figure 2). This comes at the price of more complex networks.

 Cytoscape offers nine different node shapes, and therefore it is not possible to represent all the SBGN entities based on this list alone (Figure 2a). To overcome this we use the custom node graphics feature in Cytoscape (introduced in Cytoscape 2.8 [18]), which renders any specific image above the nodes. Thus, CySBGN encompasses all the SBGN shapes by loading a previously created image in the respective type of node.

**Figure 3:**
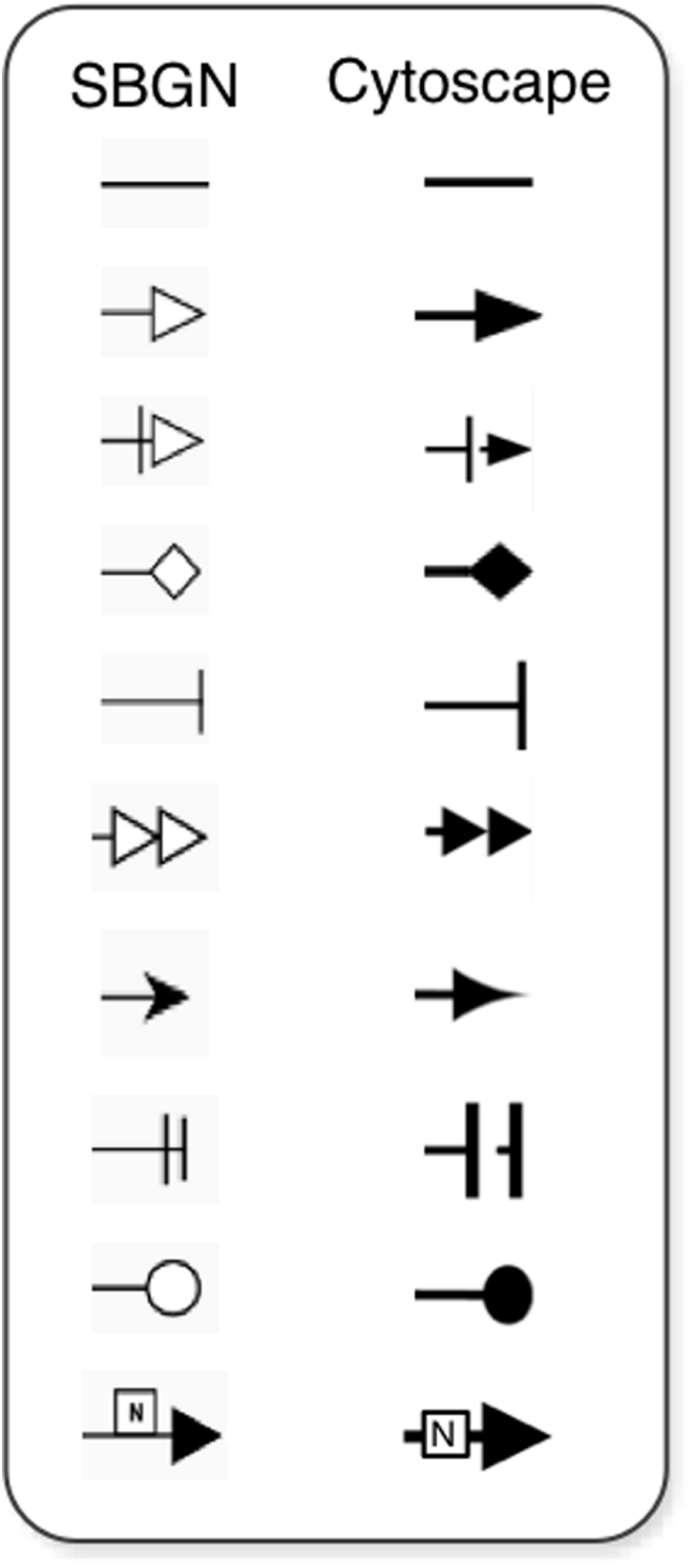
**Edges shapes comparison between SBGN and CySBGN.** Edge shapes comparison between SBGN and CySBGN. Comparison scheme of the edge shapes in the original layout of SBGN and the one adopted by CySBGN. Mostly the differences reside in the edge shape filling colour, white in SBGN and black in Cytoscape, although there are also some minor differences in some shapes.

Similarly to the node shapes, not all the relationship node shapes (i.e. edges) are directly represented in Cytoscape. In particular, absolute stimulation, necessary stimulation, and absolute inhibition relationships lack a representation (Figure 2b). We noticed that the edges that are not directly supported may be split into two sub edges, e.g. the necessary stimulation edge may be subdivided into an inhibition edge followed by a stimulation edge. Taking advantage of this, CySBGN handles all unsupported relationship nodes through a composition of two supported edges linked by a small invisible auxiliary node (Figure 2b). It should be noted that adding auxiliary nodes may affect the network topology analysis methods, since they consider these nodes for analysis even if they do not contain any biological meaning. We address this issue by allowing the user to simplify the network (more details about the network simplification method can be found in the SBGN diagram simplification section). Moreover, the edges layout is not exactly the same as the one adopted by SBGN. The differences are mostly in the filling colour of the edge’s shape, which in SBGN is white and in Cytoscape is black. A full comparison between the edge shapes of SBGN and the corresponding shapes in CySBGN is depicted in Figure 3.

In order to increase the rendering performance Cytoscape disables the rendering order of the nodes. In other words, nodes do not have a *z* coordinate, and therefore when two nodes are overlapping each other it is not possible to identify which node is in the front or back. Consequently, nodes are randomly ordered (see Figure 2c). This brings some representation problems mostly when the SBGN diagrams contain nodes that enclose other nodes, as is the case of compartments. To avoid rendering the compartment above the enclosed nodes and therefore hiding them, CySBGN draws every compartment node with transparent filling. This ensures that the enclosed nodes are visible when the compartment is not selected. However, it is not possible to guarantee the transparency of a node when it is selected.

#### SBGN diagram simplification

Implemented methods in Cytoscape are optimized to work with simple network structures similar to SIF files. Hence, we created a simplification method that becomes useful in cases when the SBGN diagram structure does not fit the Cytoscape features, such as applying a layout algorithm to a SBGN diagram with cloned entities. In SBGN, a *clone marker* (dark shading in the bottom part of the entities) identifies nodes that appear duplicated in the network. Typical examples of clone entities are ADP or ATP, because they are present in more than one reaction. In these cases the hierarchical layout is wrongly affected by the presence of the cloned entities. Another example is when a necessary stimulation arc is present: due to its unsupported shape CySBGN represents it as an aggregation of two edges (see Results and discussion section for more details). This composition of edges affects the layout analysis of the network. The simplification feature duplicates the original diagram but disregards all nodes that are not supported by Cytoscape, such as compartments, tags or cloned entities. In addition, the edges that need to be drawn as a composition of other edges (i.e necessary stimulation, absolute inhibition and absolute stimulation) are simplified into a single edge (Figure 2b). Using the simplification feature the diagram information is retained, since the edge type attribute is preserved and only the edge shape is changed. Moreover, the simplification is represented in another diagram keeping the original one unchanged. In the CySBGN tutorial (http://www.ebi.ac.uk/saezrodriguez/cysbgn/files/tutorial.pdf) an example of a simplification of a map kinase cascade diagram is provided.

#### SBML to SBGN converter

To increase the interoperability of CySBGN we developed an integrated feature between CySBGN and CySBML [13] that enables the users to automatically generate a SBGN diagram based on a SBML model. Taking advantage of the features made available by the SBFC [17], CySBGN generates a SBGN-ML file from a previously selected SBML model and consequently imports it into Cytoscape. This allows the SBML model visualization to be complemented with the respective SBGN diagram, providing the users the possibility to generate the most convenient visualisation of the model, store it, and share it among users and tools. The CySBGN tutorial (http://www.ebi.ac.uk/saezrodriguez/cysbgn/files/tutorial.pdf) provides a step-by-step guide of this feature, it also provides information on how to obtain SBML models from the BioModels database [19].

## Results and discussion

The accuracy of diagram rendering in CySBGN was validated by running a varied and extensive number of test cases containing maps from the three complementary languages of SBGN. For each case, we imported a SBGN-ML file and compared the generated diagram with the expected layout. The AF language was tested with 8 diagrams, the ER with 18 and the PD with 27, leading to a total of 53 different maps, covering almost all shapes and connection types. This suite of test cases is shared among other SBGN compliant tools, such as PathVisio [7] or SBGN-ED [20] (a VANTED [8,9] plug-in which creates and edits SBGN diagrams). Hence, it is possible to compare the results obtained by CySBGN with the original (expected) diagrams and the ones obtained in other tools.

**Figure 4:**
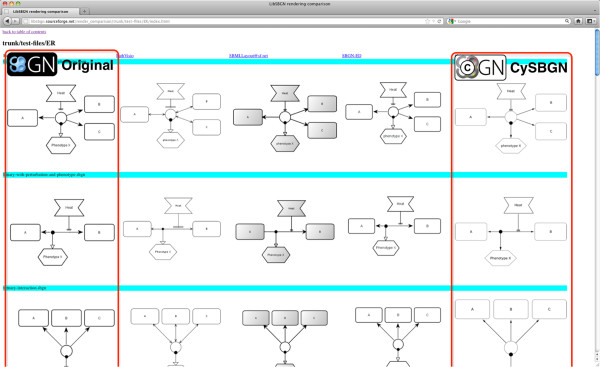
**CySBGN rendering comparison.** Rendering comparison among the tools supporting SBGN. The comparison is automatically generated by a python script and it is hosted at http://libsbgn.sourceforge.net/render_comparison/. The first column shows the original diagrams and the last column shows the rendered diagrams obtained with CySBGN. The obtained rendering in PathVisio, SBML Layout and VANTED are shown in the second, third and fourth column, respectively.

To perform automatically this comparison we developed a python script that generates a html page containing a side-by-side comparison showing the expected pictures of the SBGN maps and those obtained by CySBGN (see Figure 4). The resulting maps are displayed in a matrix structure where the columns correspond to different applications and the rows to the different test cases. The first column refers to the original and correct drawing of the map and the last column is the drawing obtained by CySBGN. Due to its large size the comparison table is available as a supplementary file (Additional file 1) and can also be visualized at http://libsbgn.sourceforge.net/render_comparison/.

CySBML allows users to import SBML models directly from BioModels database [19], visualize and analyse them. We used this feature to test the automatic generation of SBGN diagrams from the SBML models. This allows one to pick a pathway from BioModels, download the respective SBML model and then generate the SBGN diagram that can be further used as a publication diagram or simply shared with other users. The plug-in’s tutorial is available on the CySBGN web page (http://www.ebi.ac.uk/saezrodriguez/cysbgn/files/tutorial.pdf) and provides detailed step-by-step guides to all CySBGN features. Additionally, the plug-in’s applicability is illustrated by using two different Cytoscape plug-ins to apply network analysis methods to SBGN diagrams. The first example shows the identification of the shortest path between pyruvate and succinate in a large metabolic network using the CytoHubba plug-in (http://hub.iis.sinica.edu.tw/cytoHubba/). The second example demonstrates how users can identify network motifs using the NetMatch [21] plug-in. Both examples use a large SBGN diagram of the plant central metabolism (1324 nodes and 1322 edges).

Future steps in CySBGN will focus on: *(i)* further integration with other Cytoscape plug-ins (e.g. BiNoM [15] integrates a wide variety of structure analysis methods and ways to convert the CellDesigner SBML extension to BioPAX and BioPAX to SBML), and *(ii)* improving the diagram export feature to allow a full mapping of the changes made to the SBGN diagram (i.e. allow the user to add/remove entities and arcs and also allow the storage of any other modifications made to the diagram). Furthermore, we will upgrade CySBGN compatibility to the upcoming version of Cytoscape, Cytoscape 3, when it is released.

## Conclusions

Here we presented a new plug-in for Cytoscape, CySBGN, that provides support to SBGN diagrams. CySBGN allows one to load and visualize SBGN-ML models in Cytoscape and leverage all its repertoire of features and plug-ins. Among other features it also establishes a connection with the CySBML plug-in and SBFC, allowing the automated generation of SBGN diagrams of any imported SBML model. A detailed tutorial containing several step-by-step guides covering all CySBGN features is made available in the plug-in web page (http://www.ebi.ac.uk/saezrodriguez/cysbgn/files/tutorial.pdf).

Cytoscape’s active community allows the development of the plug-ins to be processed smoothly. Thus, features like extending Cytoscape’s supported edges and nodes shapes may be added in near future, consequently improving CySBGN. Moreover, there is an increasing number of plug-ins available [22], thus we expect more synergies of CySBGN with other plug-ins in the future.

## Availability and requirements

•**Project name:** CySBGN

•**Project home page:**http://www.ebi.ac.uk/saezrodriguez/cysbgn/

•**Operating system(s):** Platform independent

•**Programming language:** Java

•**Other requirements:** Java 1.6 or higher, Cytoscape 2.8.3

•**License:** GNU GPL v3

•**Any restrictions use by non-academics:** Only those imposed already by the license

## Competing interests

The authors declare that they have no competing interests.

## Authors’ contributions

JSR and MVI proposed the idea, initiated the project and evaluated the software by pointing out errors and improvements. EG and MVI designed the software architecture. EG implemented CySBGN. EG and JSR wrote the paper. All authors read and approved the final manuscript.

## Supplementary Material

Additional file 1 **Supplementary materials 1 CySBGN rendering validation and comparison.** Exhaustive multi-page table containing the rendering comparison of CySBGN with other SBGN compliant tools and the original drawing of the 53 validation test cases proposed in the manuscript. Click here for file
