# Supplementary material for: CySBGN: A Cytoscape plug-in to integrate SBGN maps
Source: BMC Bioinformatics. 2013 Jan 16;14:17. doi: 10.1186/1471-2105-14-17 (PMC3599859; doi:10.1186/1471-2105-14-17)
Supplement: Additional file 1 — Supplementary materials 1 CySBGN rendering validation and comparison. Exhaustive multi-page table containing the rendering comparison of CySBGN with other SBGN compliant tools and the original drawing of the 53 validation test cases proposed in the manuscript. [file 1471-2105-14-17-S1.PDF]

back to table of contents

trunk/test-files/AF

Reference | PathVisio | SBMLLayout@sf.net | SBGN-ED | Cytoscape

auxiliary-units.sbgn

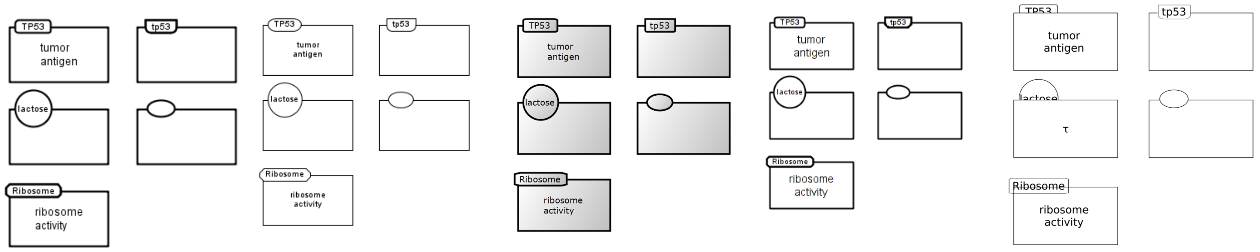

activity-nodes.sbgn

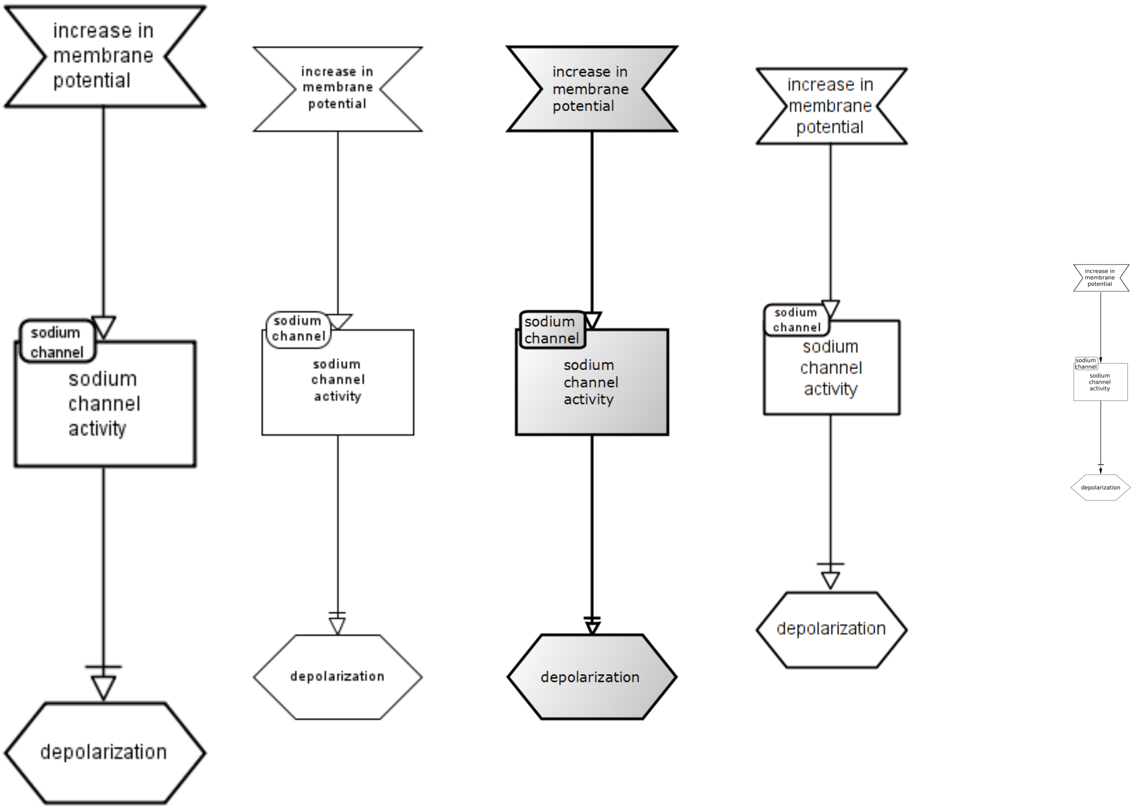

submap_expanded.sbgn

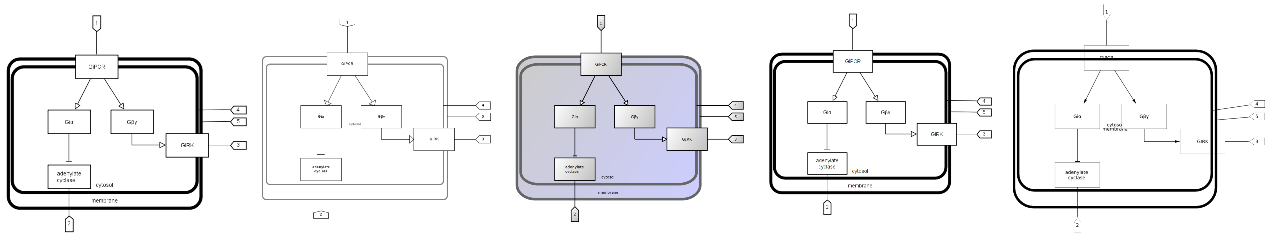

modulation.sbgn

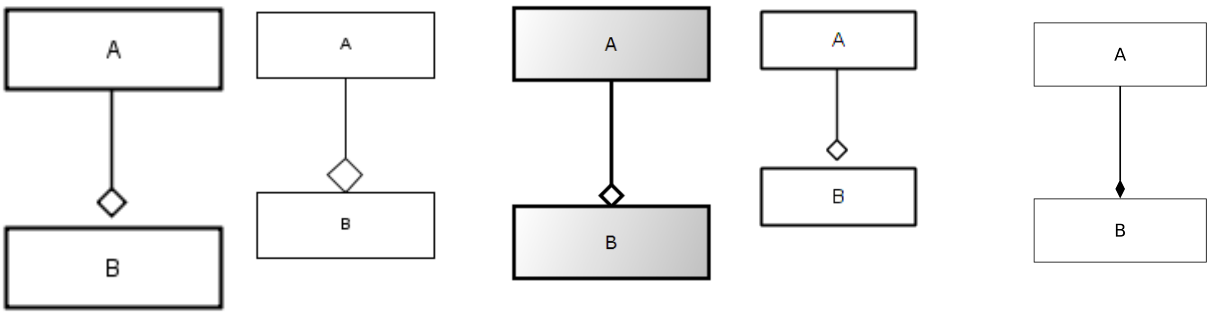

submap.sbgn

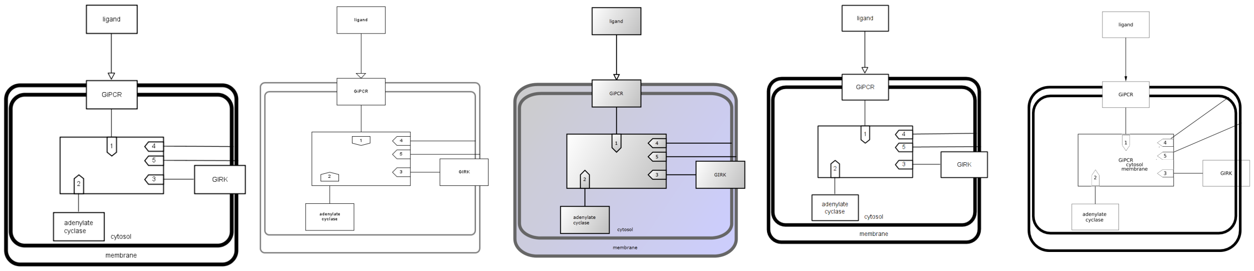

two_edges_between_two_activities.sbgn

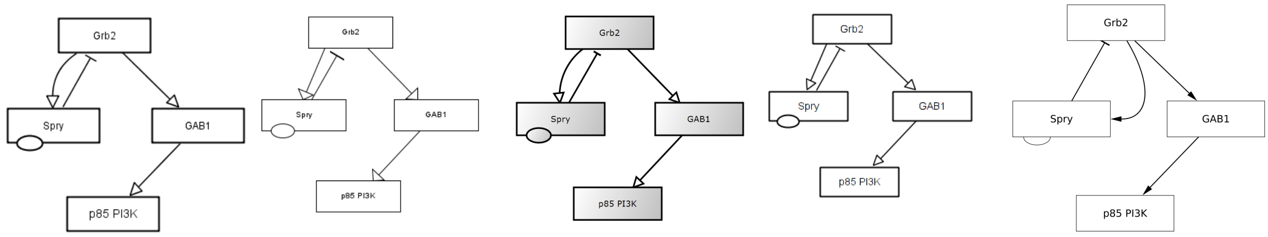

compartment.sbgn

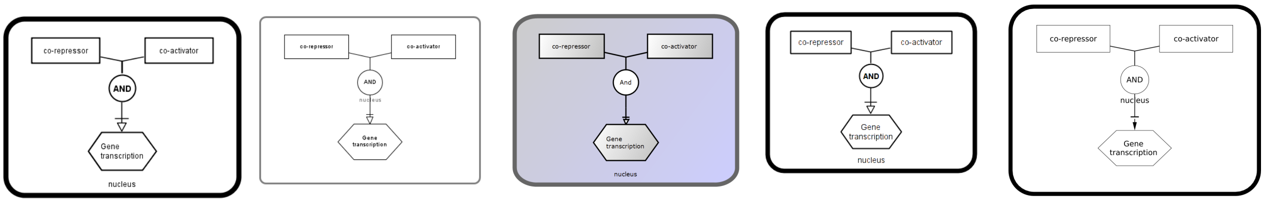

delay.sbgn

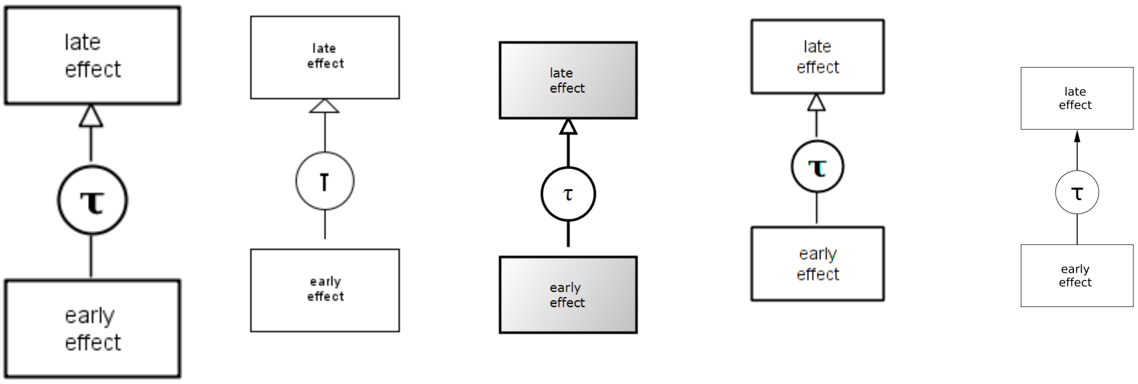

LibSBGN rendering comparison created by Martijn van Iersel
Generated: 2012-05-02

[back to table of contents](#)

trunk/test-files/ER

Reference	PathVisio	SBMLLayout@sf.net	SBGN-ED	Cytoscape

ternary-with-perturbation-and-phenotype.sbgn

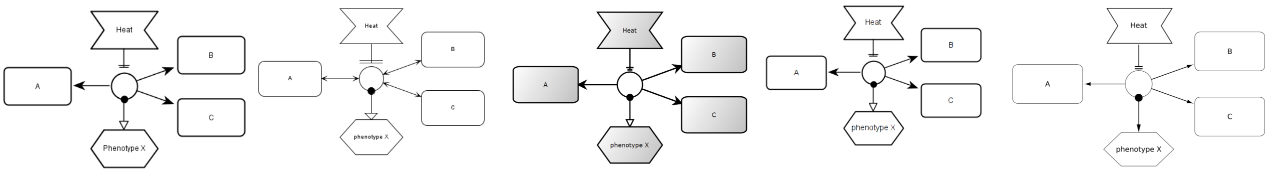

binary-with-perturbation-and-phenotype.sbgn

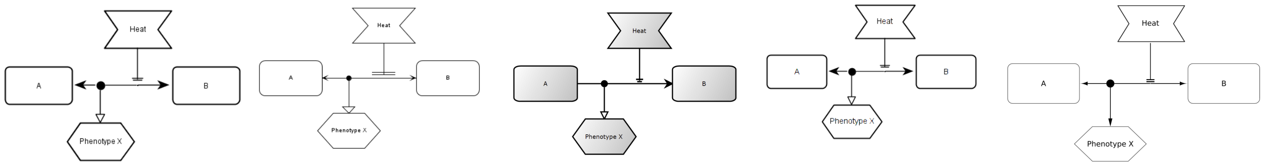

ternary-interaction.sbgn

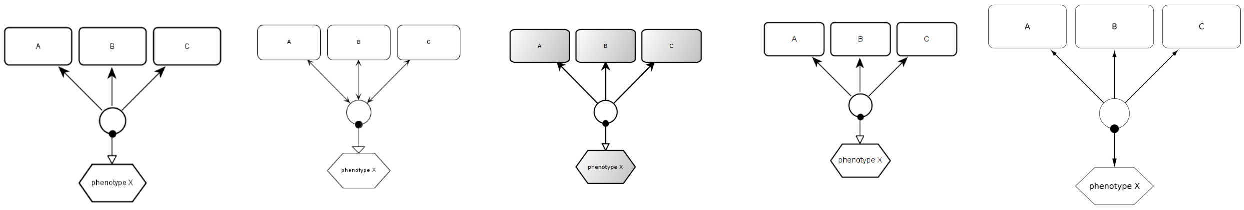

binary-no-outcome.sbgn

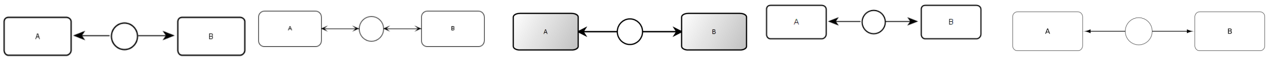

implicit_xor.sbgn

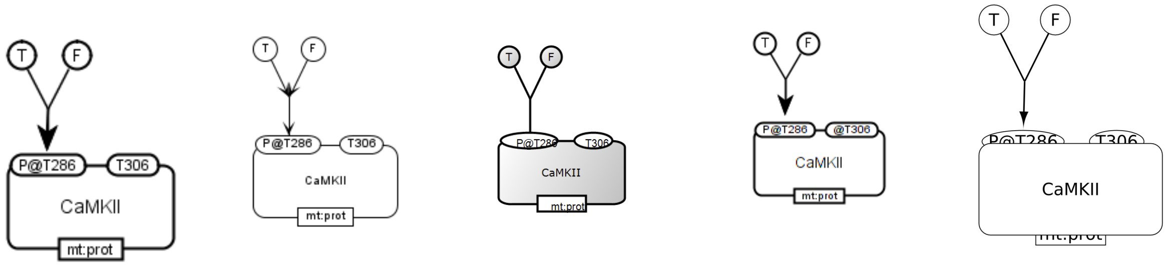

ternary-with-cardinality.sbgn

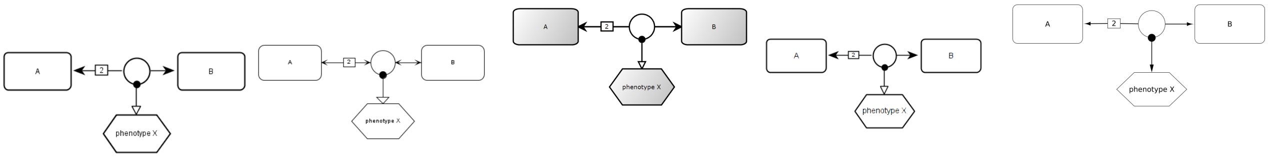

existence_variable.sbgn

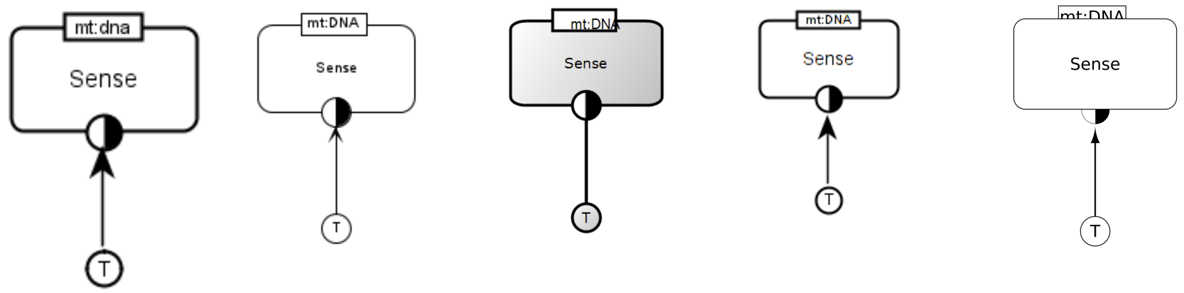

phosphorylation.sbgn

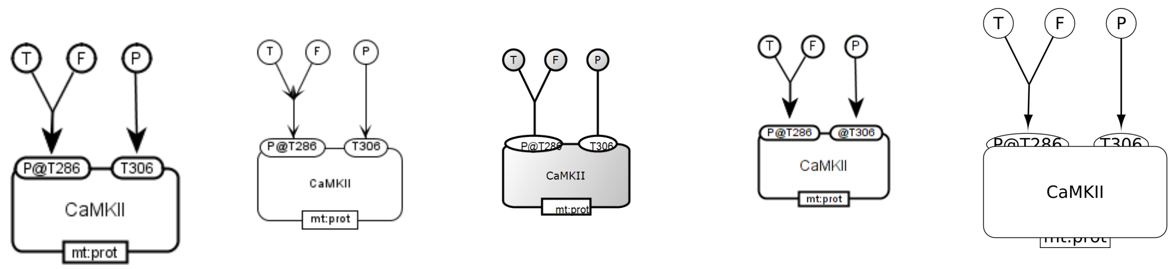

absolute inhibition.sbgn

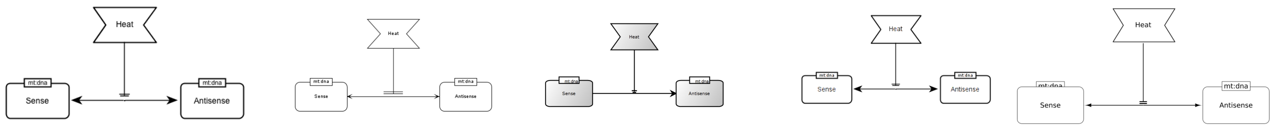

cis.sbgn

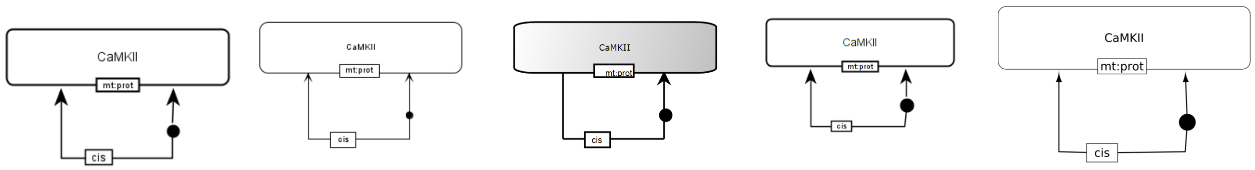

absolute stimulation.sbgn

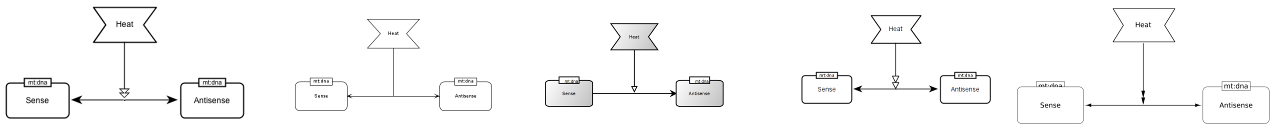

OR gate.sbgn

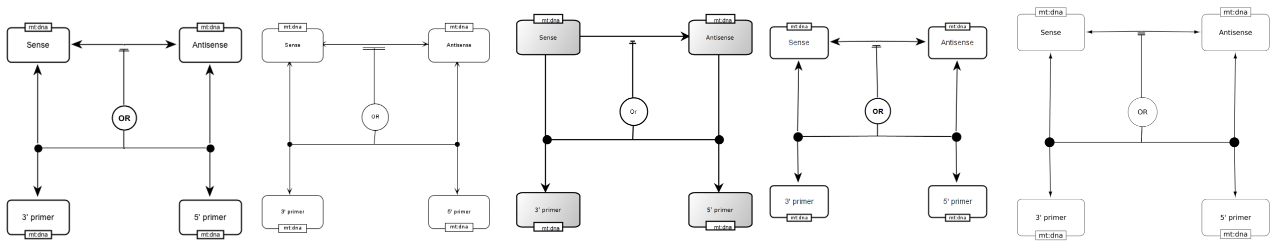

outcome-simplified.sbgn

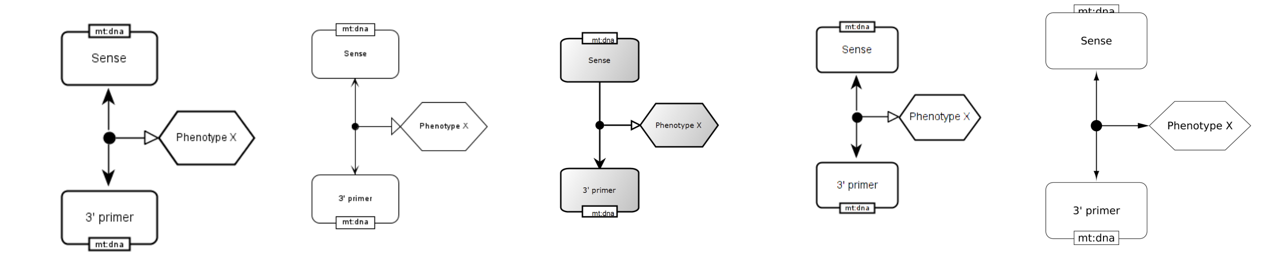

trans.sbgn

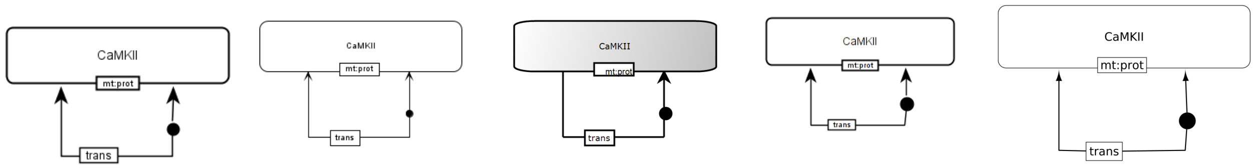

location.sbgn

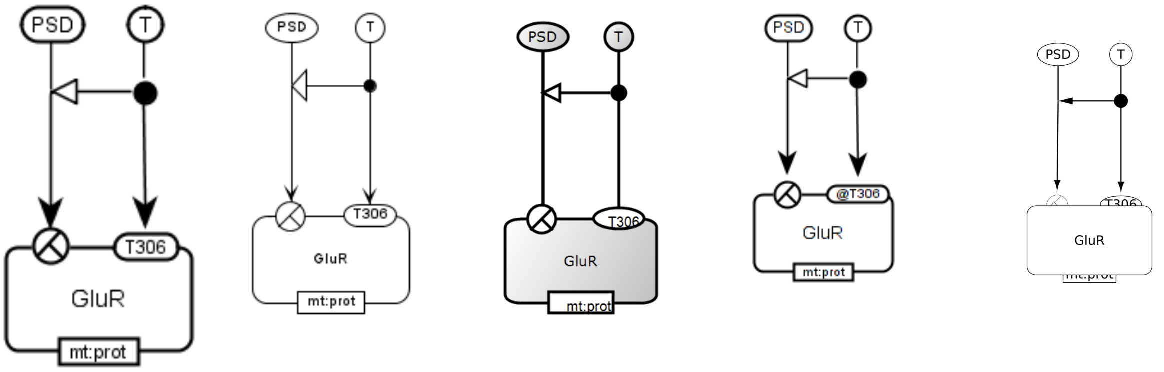

outcome-dual.sbgn

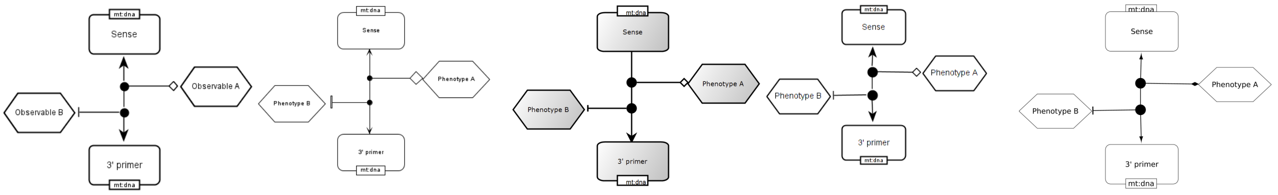

invisible node.sbgn

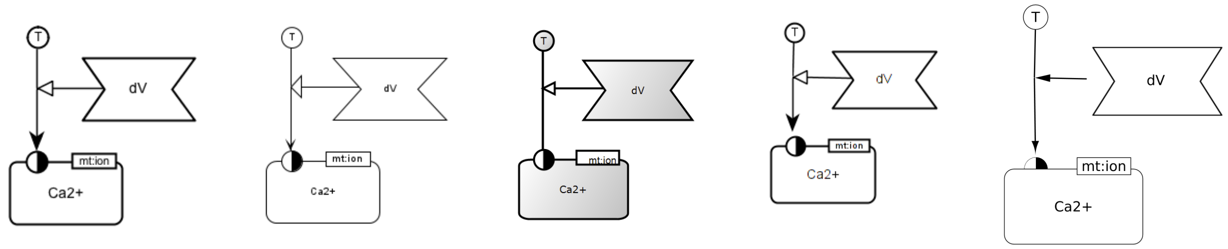

delay.sbgn

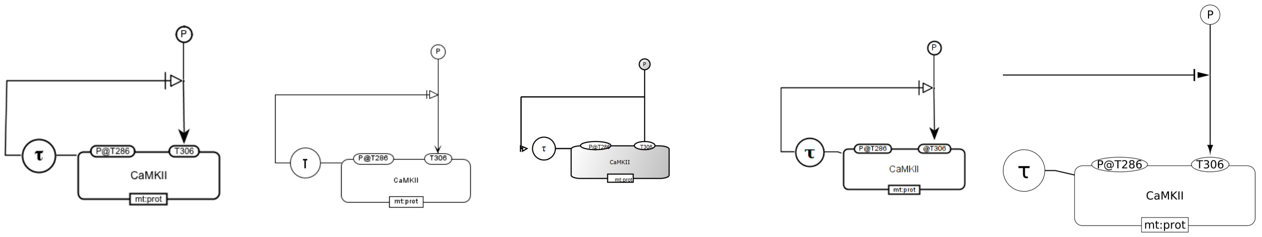

LibSBGN rendering comparison created by Martijn van Iersel
Generated: 2012-05-02

back to table of contents

trunk/test-files/PD

Reference	PathVisio	SBMLLayout@sf.net	SBGN-ED	Cytoscape

states.sbgn

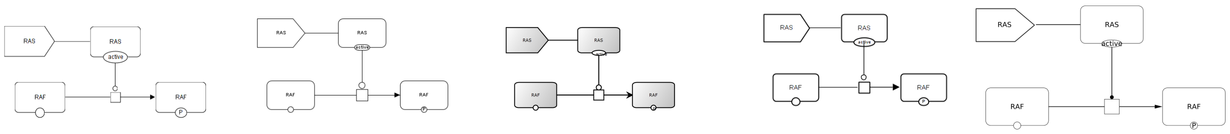

activated_stat1alpha_induction_of_the_irf1_gene.sbgn

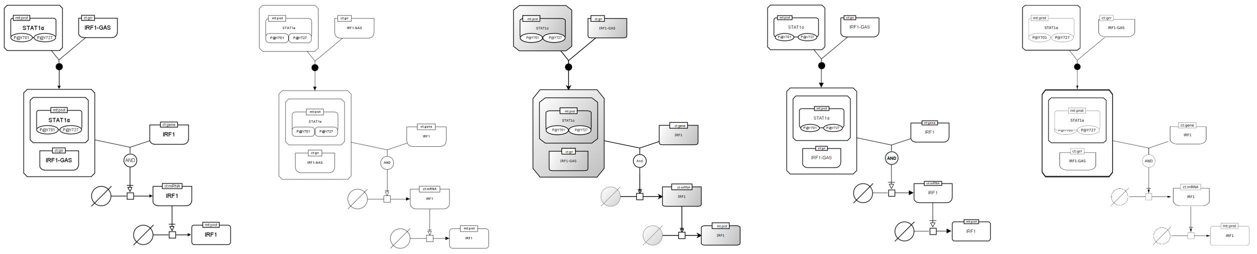

mapk_cascade.sbgn

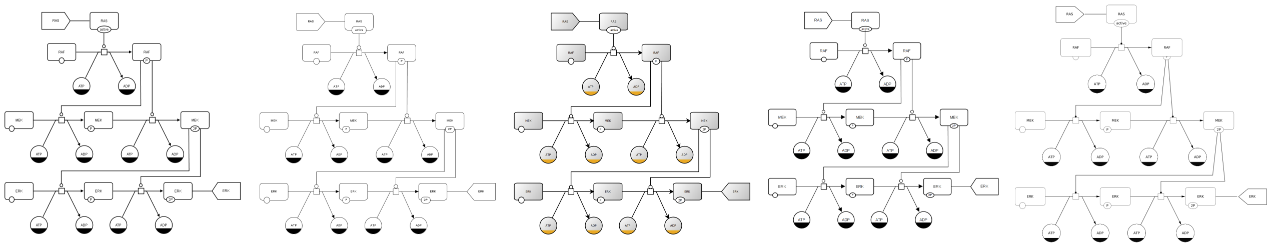

edgerouting.sbgn

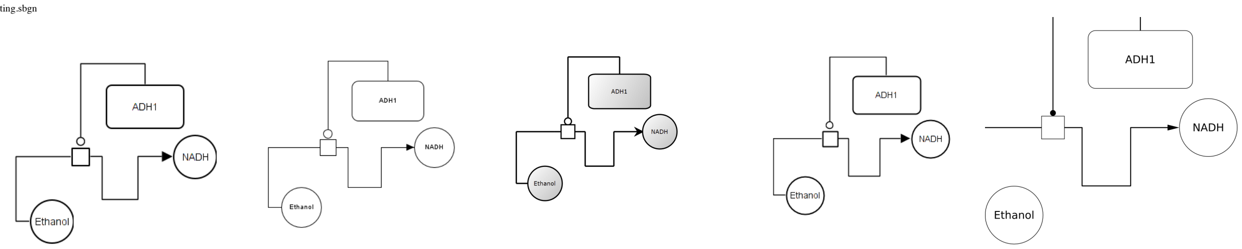

compartmentOrder2.sbgn

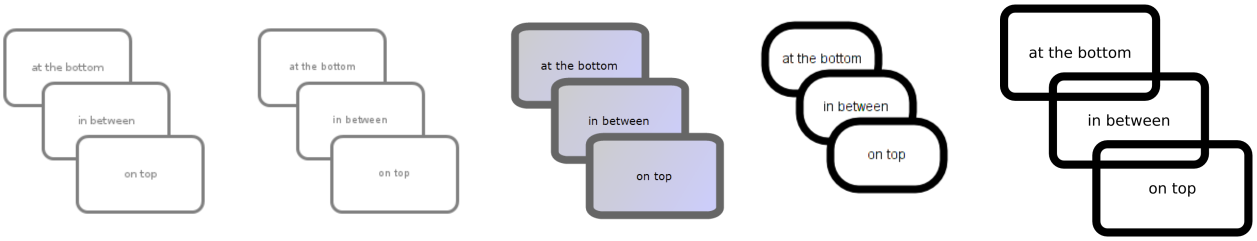

clone-marker.sbgn

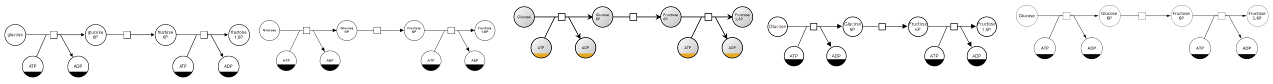

utf8_test_case_with_byte_order_mark.sbgn

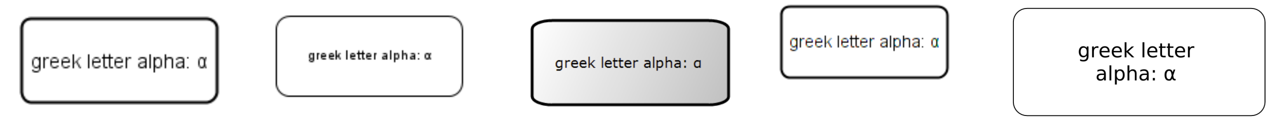

multimer2.sbgn

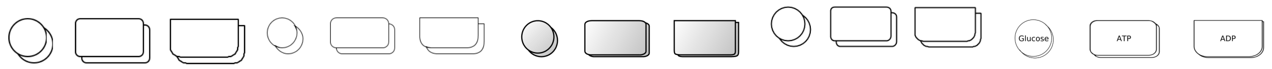

and.sbgn

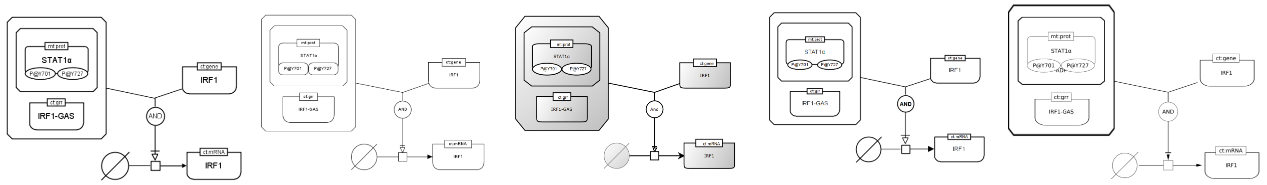

stoichiometry.sbgn

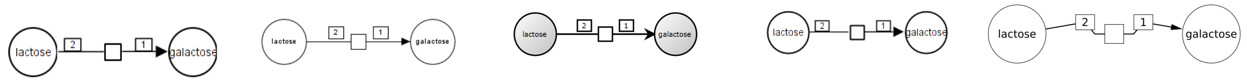

protein_degradation.sbgn

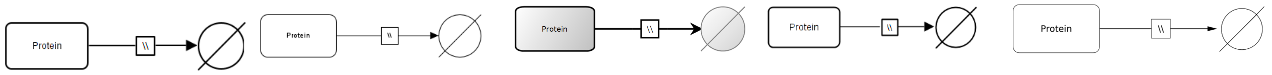

insulin-like_growth_factor_signaling.sbgn

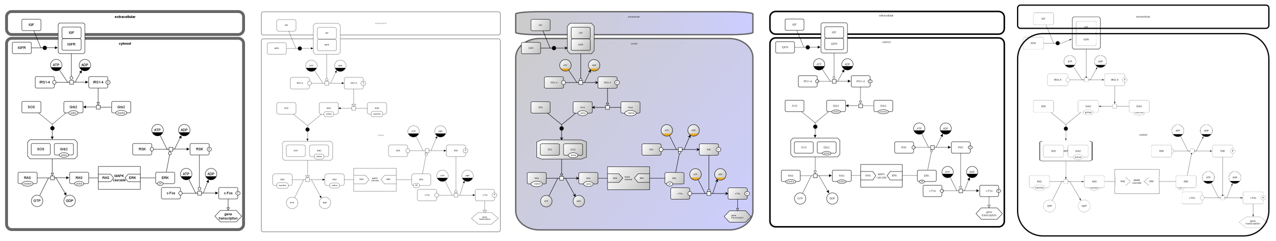

compartmentOrder1.sbgn

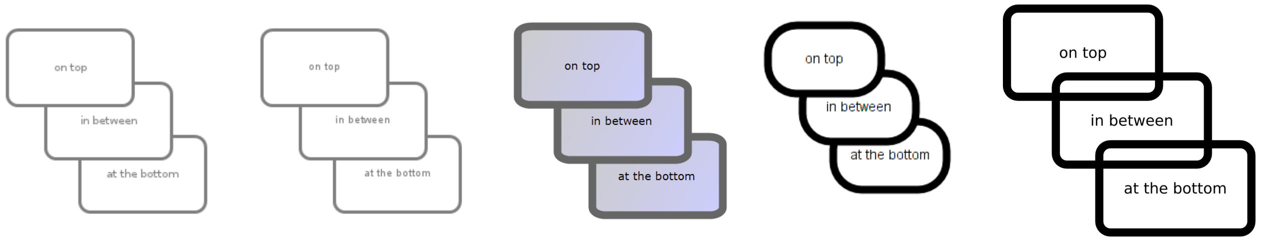

adh.sbgn

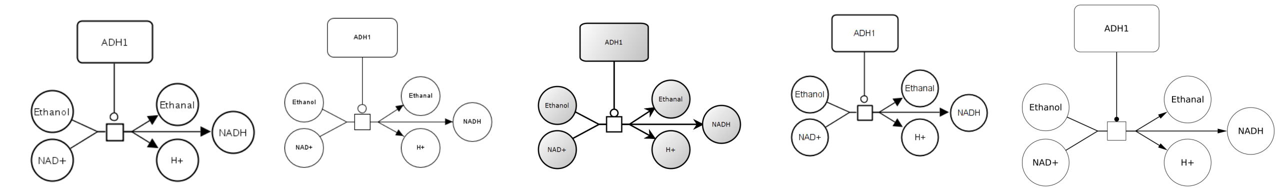

multimer.sbgn

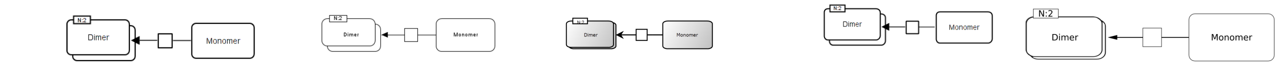

neuronal_muscle_signalling_color.sbgn

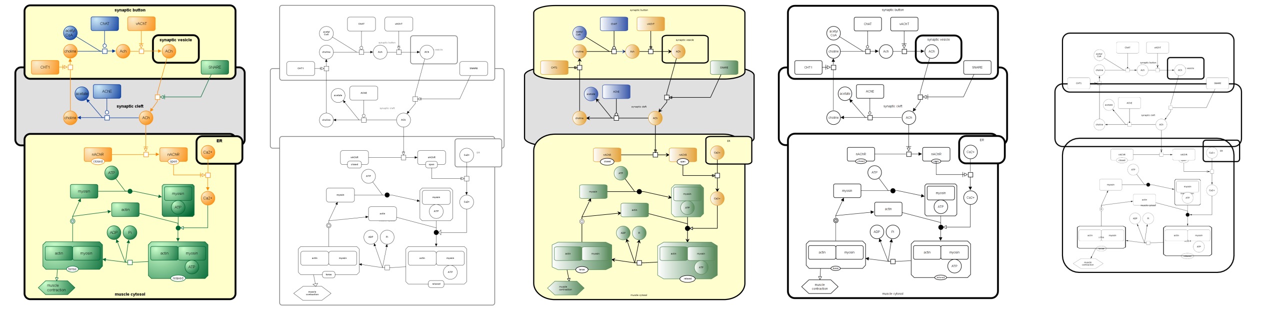

annotation.sbgn

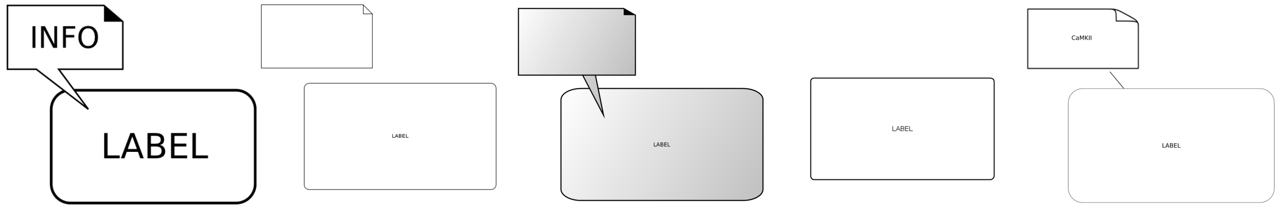

reversible-verticalpn.sbgn

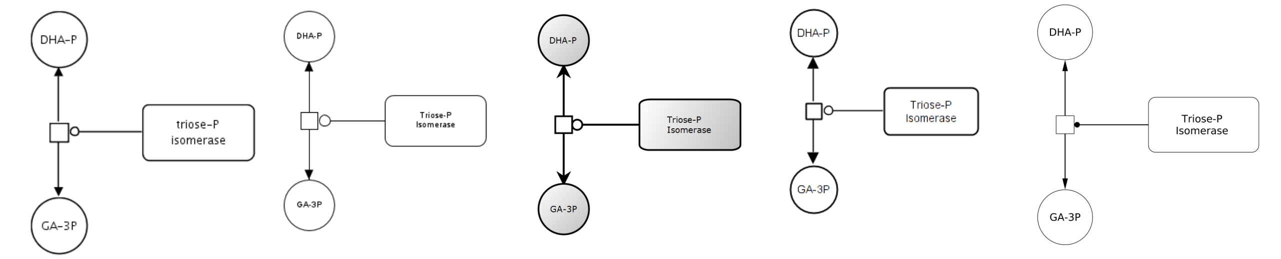

or-simple.sbgn

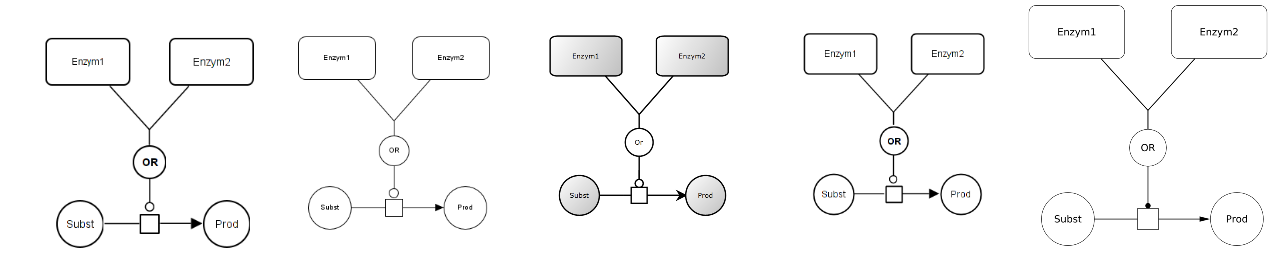

bool-expr-pd.sbgn

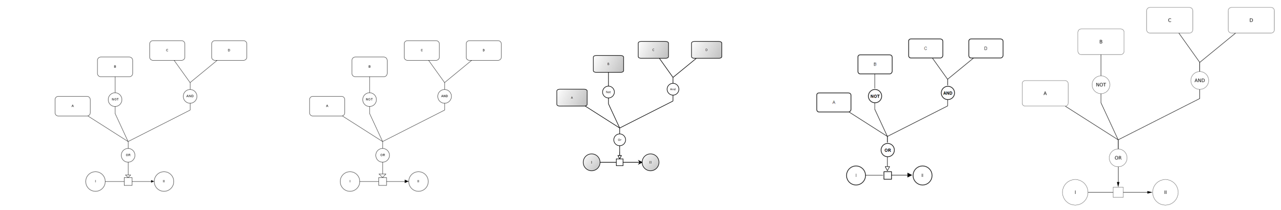

glycolysis.sbgn

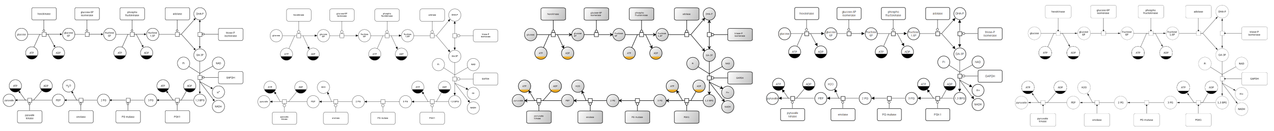

statesType2.sbgn

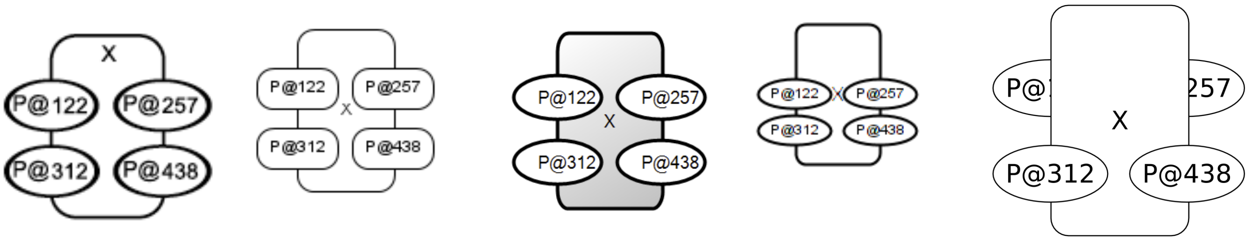

neuronal_muscle_signalling.sbgn

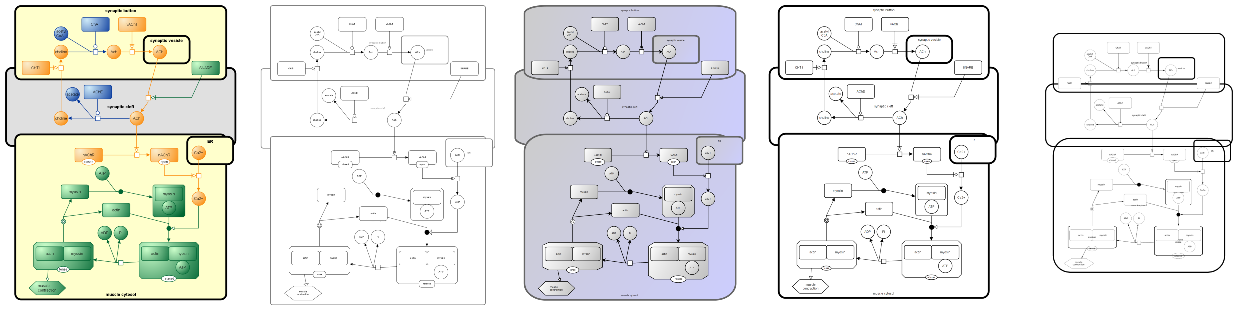

compartments.sbgn

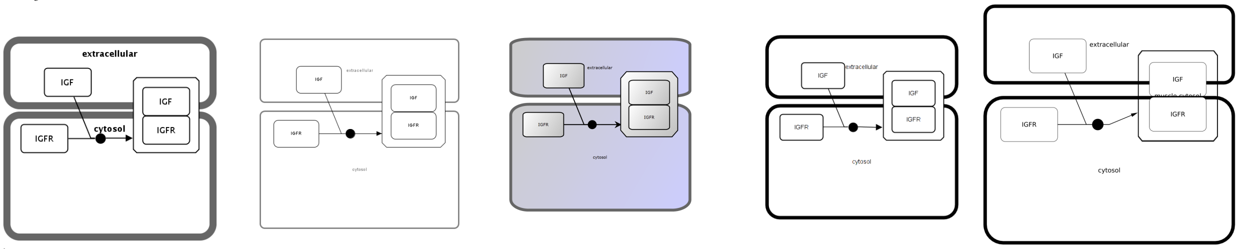

submap.sbgn

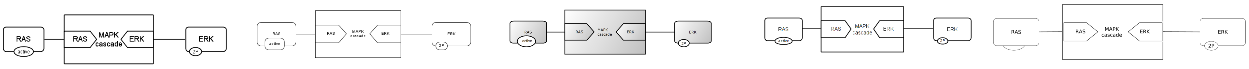

labeledCloneMarker.sbgn

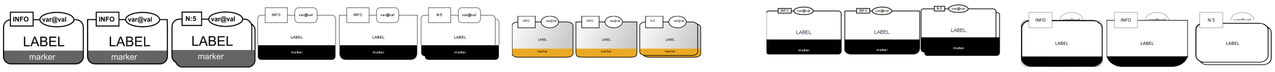

utf8_test_case_without_byte_order_mark.sbgn

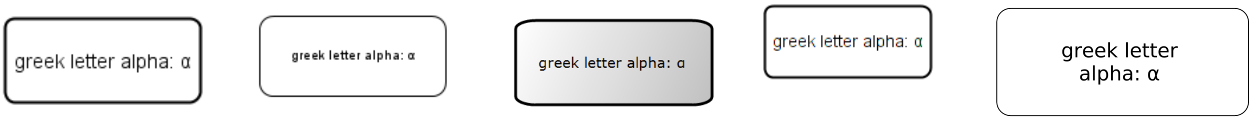

LibSBGN rendering comparison created by Martijn van Iersel

Generated: 2012-05-02
